# Artificial intelligence in respiratory research: opportunities, pitfalls, and ethical considerations

**DOI:** 10.36416/1806-3756/e20240346

**Published:** 2024-12-17

**Authors:** Ricardo G Figueiredo, Juan Calderón

**Affiliations:** 1. Programa de Pós-Graduação em Saúde Coletiva, Universidade Estadual de Feira de Santana - PPGSC-UEFS - Feira de Santana (BA) Brasil.; 2. Fundação ProAR, Salvador (BA) Brasil.; 3. Methods in Epidemiologic, Clinical, and Operations Research-MECOR-program, American Thoracic Society/Asociación Latinoamericana del Tórax, Montevideo, Uruguay.; 4. Universidad Espíritu Santo, Samborondón, Ecuador.; 5. Respiralab Research Group, Guayaquil, Ecuador.

The rapid advancement of technology in science is genuinely remarkable. The scientific community has been shocked by artificial intelligence’s (AI) transformative potential in research, leveraging its generative capabilities through artificial neural networks, machine-learning algorithms, and large language models (LLMs), enabling them to analyze complex databases and mimic the human brain’s data-processing functions.^1^

There is also great interest in AI being employed to elucidate underlying mechanisms of diseases and identify optimal treatments by detecting disease patterns and analyzing large databases.^2^ ^,^ ^3^ Big Data in medical research represents a transformative shift in how researchers collect, analyze, and apply these data to improve patient outcomes, understand diseases better, and enhance healthcare delivery. This revolution is driven by the exponential growth of data from various sources, including electronic health records, genomic sequencing, and wearable health devices. Machine learning algorithms can integrate data in a multidimensional analysis, including genomics, metabolomics, social, environmental, and health records. Specifically in the respiratory field, AI could interpret pulmonary function tests as accurately as pulmonologists.^4^ It can also assist in diagnosing small airway diseases by utilizing more advanced and complex methods such as oscillometry.^5^ We are witnessing the beginning of a new era in respiratory care that empowers physicians, enhances diagnostic accuracy, and improves patient safety. Nevertheless, in daily medical practice, misclassification of diagnoses can have life-or-death consequences, making it essential to monitor the development and application of this technology closely. Quoting Prof. Judith Löffler-Ragg: “It is extremely important that we approach technological advancements, particularly AI, with both an open mind and a critical eye.”

Ideally, AI would alleviate researchers from the burden of bureaucratic tasks, enhance human creativity, break down language barriers, and enable a shared control dynamic while humans retain responsibility and accountability. As generative AI continually improves through the increasing availability of data and user feedback, its impact on compliance with research methodology, data analysis, and the integrity of academic publishing becomes increasingly significant. However, biased data and models used to train AI systems can result in flaws being reflected in the final models or reports generated by AI, which may compromise the accuracy and reliability of the outcomes. This reflects the principle of “garbage in, garbage out,” where flawed input inevitably leads to flawed results. Researchers should also be aware that LLMs, such as ChatGPT and Google Gemini, can hallucinate. This means that generative AI may produce highly convincing text with entirely incorrect concepts and even fabricate references that do not exist. This implies that any information produced by AI technologies must be carefully reviewed by a human and appropriately reported. There is growing concern that AI could contribute to a reproducibility crisis in science by fostering the proliferation of low-quality research.

An analysis of the Scopus database suggests that the proportion of research papers with titles or abstracts mentioning AI or machine-learning terms has increased to approximately 8%. The survey conducted by Van Noorden et al.,^6^ involving over 1,600 researchers globally, revealed mixed feelings about the increasing role of AI in research. While most respondents recognized the potential benefits, such as faster data processing and the ability to approach previously infeasible research questions, significant concerns were raised. Major issues included the risk of AI systematic biases, making plagiarism easier, and introducing inaccuracies into research. The survey also highlighted that many researchers remain cautious about their broader adoption in scientific workflows.

Respiratory research funding is inadequate and inequitable, with a significant gap between the disease burden and research investment.^7^ This disparity is especially pronounced among researchers from developing countries and non-native English speakers, who face additional obstacles in global research participation and recognition. Discrimination, exclusion, and stereotyping extend beyond data collection are embedded in societal inequalities, influencing how data is processed and classified.^8^ Grammarly and ChatGPT offer valuable support to these researchers by helping them improve their writing style, clarity, and coherence of the manuscript. Generative AI tools can assist non-native speakers to engage more effectively with the global scientific community. Scite is an AI tool that employs LLMs to search academic literature using natural, plain language. It verifies the accuracy of references used to support ideas in a manuscript and alerts users to errata or retractions. This tool can be especially helpful for early-career researchers during the literature review process, helping them refine their research before conceiving a final PICO (Patients of interest, Intervention to be studied, Comparison of intervention, and Outcome of interest) question.

Unfortunately, not everything that glitters is gold. While most editors and researchers acknowledge the increasing use of AI in scientific publishing, numerous reports of potential misuses in science have raised significant red flags. A case study involving the publishing process of an entirely AI-generated manuscript brings several concerns.^9^ ChatGPT 3.5 can convincingly produce fabricated references that do not exist, raising concerns about the reliability and integrity of AI-generated academic content. The manuscript above was accepted by six of twelve submissions and provisionally accepted in another journal. Although editorial offices can screen incoming manuscripts for AI-generated content using GPTZero or Originality.AI, these tools currently cannot differentiate between the legitimate use of AI for grammar and writing enhancement and fully AI-generated text. This limitation poses challenges in accurately assessing the extent of AI involvement in manuscript preparation.

Several ethical issues have been raised regarding the use of AI in manuscript preparation, leading to swift policy adjustments in academic publishing. In recent years, the International Committee of Medical Journal Editors (ICMJE) developed recommendations that permit AI use but explicitly prohibit AI from being listed as an author, as it cannot take responsibility for the accuracy, integrity, or originality of the research.^10^ Additionally, AI involvement must be disclosed, and authors are also responsible for ensuring the absence of plagiarism, including in AI-generated text and images, and must adequately attribute all quoted material with proper citations.

Plagiarism is a critical concern in scientific publishing. As hybrid human-AI writing becomes more common, the distinction between human and AI contributions may become increasingly difficult. Ideally, AI would augment human creativity, remove language barriers, and assist in various tasks while humans maintain control over the final output.^11^ Although researchers can delegate aspects of writing to AI tools, they remain fully accountable for the content’s accuracy, integrity, work originality, and adherence to ethical standards. The growing significance of AI in medicine has motivated the JAMA Network to introduce a dedicated section titled JAMA+ AI, which emphasizes the impact of AI on healthcare.^12^

While AI can be utilized throughout various stages of research-from formulating research questions to preparing manuscripts-,it should primarily be employed to enhance processes in research workflow, pattern recognition and trend analysis (“how”). However, the irreplaceable human capacities for creativity and critical thinking are essential for tackling more complex scientific questions and addressing uncertainties (“why”). These abilities are even more crucial in an era of advanced AI. We are now at a pivotal moment where it is essential to clearly define the areas of research and society in which AI can be safely integrated and determine the most effective strategies for its implementation. A comprehensive understanding of AI’s potential risks and challenges is crucial to ensuring its responsible use in research.
